# Biotechnological and Biomedical Applications of Enzymes Involved in the Synthesis of Nucleosides and Nucleotides—2nd Edition

**DOI:** 10.3390/biom14111438

**Published:** 2024-11-12

**Authors:** Jesús Fernández-Lucas

**Affiliations:** 1 Applied Biotechnology Group, Universidad Europea de Madrid, Urbanización El Bosque, Villaviciosa de Odón, 28670 Madrid, Spain; jesusf08@ucm.es; 2 Grupo de Investigación en Ciencias Naturales y Exactas, GICNEX, Universidad de la Costa, CUC, Calle 58 # 55-66, Barranquilla 080002, Colombia; 3 Department of Biochemistry and Molecular Biology, Faculty of Biology, Universidad Complutense de Madrid, C. de José Antonio Novais, 12, 28040 Madrid, Spain

The study of nucleic acid derivatives and their role in cellular processes, first addressed in our previous Special Issue (https://www.mdpi.com/journal/biomolecules/special_issues/nucleotides_applications), continues to be a focal point of significant scientific exploration. In the earlier collection, we examined the crucial pathways—de novo and salvage—for nucleotide synthesis, along with the enzymes involved in these processes and their importance for chemotherapy and biocatalysis. Building on this foundation, this Special Issue provides an in-depth exploration of recent advances in the biotechnological and biomedical applications of enzymes involved in nucleoside and nucleotide synthesis, emphasizing novel insights into their therapeutic potential, biochemical functions, and emerging applications in synthetic biology.

To this end, a total of six articles, including five experimental studies and one review article, authored by world-leading experts have been compiled to showcase the state-of-the-art advancements in this field.

As anticipated, among the numerous studies focusing on transglycosylation reactions catalyzed by glycosyltransferases [1,2,3,4], the enzyme-mediated synthesis of nucleoside analogs (NAs) using phosphorylases (NPs) represents a significant milestone in this Special Issue. For instance, Bycheck et al. delve into the enzymatic synthesis of nucleoside analogs utilizing purine nucleoside phosphorylases (PNPs). They compare the substrate specificity of PNPs from both mesophilic and thermophilic bacterial sources, aiming to illuminate the key advantages and limitations of enzyme-based synthesis. Their article offers valuable insights into overcoming challenges related to substrate specificity, solubility, and reaction conditions. Similarly, Stachelska-Wierzchowska and Wierzchowski review the chemo-enzymatic synthesis of highly fluorescent nucleoside analogs, highlighting their potential applications in analytical biochemistry and cell biology. In this context, they detail the synthesis and properties of fluorescent ribofuranosides produced with PNPs as catalysts, presenting innovative methods for enzyme assays and cellular imaging that expand the toolkit available to researchers in this field.

This Special Issue also features two novel research articles focused on the use of nucleoside phosphorylases (NPs) as catalysts for synthesizing NAs. In the first article, Fateev et al. investigate the synthesis of substituted 1,2,4-triazole-3-thione nucleosides from mono- and disubstituted 1,2,4-triazole-3-thiones using bacterial PNPs. They also compare the activity of these triazole bases and their corresponding nucleosides against a herpes simplex virus model. Their results demonstrate that introducing thionic (-SR) and bulky aromatic substituents at the C3 and C5 positions significantly affects the bioavailability and biological activity of the 1,2,4-triazole nucleosides. In a second article, enzymatic transglycosylation processes involved in synthesizing 8-aza-7-deazapurine fleximer nucleosides using recombinant *E. coli* PNP is addressed. Interestingly, the authors focus on synthesizing and characterizing minor products, providing new insights into the reaction mechanisms and substrate interactions.

Beyond the application of glycosyltransferases, a research article on the synthesis of chiral acyclic pyrimidine nucleoside analogs with complementary stereochemistry using DHAP-dependent aldolases is presented. In this study, three DHAP-dependent aldolases are utilized as biocatalysts, with various pyrimidyl acetaldehydes serving as acceptor substrates, yielding new acyclic nucleoside analogs with two stereocenters and conversion rates ranging from 70% to 90%. Additionally, molecular docking analyses are conducted to provide insights into the observed diastereomeric excess.

Last but not least, an experimental article focused on the biomedical applications of 2′-deoxyribosyltransferases in cancer treatment is also presented. Building on previous findings of 2′-deoxyribosyltransferase (NDT) in suicide gene therapy, Perez et al. describe, for the first time, an immobilized-directed enzyme prodrug therapy (IDEPT) using His-*Lm*PDT immobilized on magnetic nanoparticles (PDT-MIONPs) for prodrug activation. Notably, PDT-MIONP derivatives show activity across a broad range of nucleosides, extending beyond 2′-deoxy-2-fluoroadenosine (dFAdo) to other purine prodrugs. Additionally, following the biophysical characterization of PDT-MIONP derivatives and the investigation of intracellular uptake in both tumor and non-tumor cells, the selectivity of the PDT-MIONP/dFAdo IDEPT system is tested on HeLa cells, leading to a significant reduction in tumor cell survival.

Finally, we would like to thank all the authors, reviewers, and the Editorial Board Members of *Biomolecules* for their considerable contributions to supporting the implementation of this Special Issue, and we hope that the readers will enjoy their work.
